# Learning Curve and Early Outcomes of Thoracoscopic Esophageal Atresia Repair in an Emerging Pediatric Minimally Invasive Surgery (MIS) Program: A Cumulative Sum Analysis

**DOI:** 10.3390/children13101309

**Published:** 2026-09-26

**Authors:** Andreea Moga, Patricia Cîmpeanu, Laura Bălănescu, Radu Bălănescu

**Affiliations:** 1 Department of Pediatric Surgery, “Carol Davila” University of Medicine and Pharmacy, 050474 Bucharest, Romania; andreea.moga@umfcd.ro (A.M.); laura.balanescu@umfcd.ro (L.B.); radu.balanescu@umfcd.ro (R.B.); 2 Department of Pediatric Surgery, “Grigore Alexandrescu” Emergency Hospital for Children, 011743 Bucharest, Romania

**Keywords:** esophageal atresia, tracheoesophageal fistula, thoracoscopy, minimally invasive surgery, learning curve, CUSUM, anastomotic leak, long-gap

## Abstract

**Highlights:**

**What are the main findings?**

- CUSUM analysis identified a change in operative-time trajectory at approximately the 15th case for thoracoscopic EA/TEF repair in a low-volume, emerging MIS program, with operative time falling from 3.4 to 2.5 h between the early and proficient phases.
- Long-gap thoracoscopic repair carried a significantly higher stricture rate (37.5% vs. 0%; *p* = 0.017) and longer hospital stay (66 vs. 30 days; *p* = 0.005), while leak and mortality rates did not differ significantly from open repair.

**What are the implications of the main findings?**

- Centers performing fewer than 5 cases per year should anticipate a longer learning curve than the 10 cases reported by high-volume centers and should plan for case concentration, institutional protocol maturation and mentorship rather than surgeon dexterity alone.
- Thoracoscopic traction preserved the native esophagus in all long-gap patients without esophageal replacement, supporting this strategy even in developing MIS programs—provided families are counselled about the stricture and dilatation burden.

**Abstract:**

Background: Thoracoscopic repair of esophageal atresia with tracheoesophageal fistula (EA/TEF) remains technically challenging due to the rarity of these procedures and the demanding intracorporeal suturing within the confined neonatal thorax. The aim of this study is to report the learning curve and early outcomes during the transition from open to thoracoscopic EA/TEF repair at a single center, evaluated using the cumulative sum (CUSUM) method. Methods: We retrospectively reviewed data of 79 consecutive EA/TEF patients operated on between January 2015 and June 2026. Twenty-eight underwent thoracoscopic repair and 51 open repairs. The open cohort served as a historical institutional comparator, reflecting the standard of care prior to the adoption of thoracoscopic technique. The thoracoscopic group was stratified into three learning curve phases: Early (*n* = 9), Intermediate (*n* = 9), and Proficient (*n* = 10). Anastomotic leak was defined by contrast extravasation on postoperative esophagogram. Complications were graded using the Clavien–Dindo classification. Results: The mean operative time was 2.9 ± 1.2 h (thoracoscopic) vs. 2.3 ± 1.2 h (open; *p* = 0.036). CUSUM analysis demonstrated a learning curve of approximately 15 cases, with operative time decreasing from 3.4 h (Phase 1) to 2.5 h (Phase 3; *p* = 0.034). Anastomotic leak occurred in 9/28 (32.1%) vs. 7/51 (13.7%; *p* = 0.137; underpowered comparison). Within the thoracoscopic group, long-gap patients (*n* = 8) had significantly higher stricture rates (37.5% vs. 0%; *p* = 0.017) and longer hospital stays (66 vs. 30 days; *p* = 0.005). The three long-gap patients with stricture required 11 total endoscopic dilation sessions. No statistically significant difference in mortality was observed (7.1% vs. 5.9%; *p* = 1.000). Conclusions: Thoracoscopic EA/TEF repair is feasible in an emerging MIS program with acceptable complication rates. The CUSUM-based learning curve was 15 cases. Long-gap thoracoscopic repair carries significantly higher stricture risk.

## 1. Introduction

The first thoracoscopic repair of esophageal atresia (EA) was performed in 1999 by Lobe et al. [1] and the thoracoscopic repair of EA with tracheoesophageal fistula (TEF) was first reported one year later by Rothenberg [2]. Esophageal atresia is classified according to the Gross system: type A (pure atresia without fistula), type B (proximal fistula), type C (distal fistula, accounting for approximately 85% of cases), type D (double fistula), and type E (H-type fistula without atresia). The management of long-gap esophageal atresia poses particular challenges, as the distance between the esophageal segments precludes primary anastomosis and necessitates either delayed reconstruction techniques such as internal traction (Foker) or, historically, esophageal replacement with gastric, colonic, or jejunal conduits. Since then, many pediatric surgeons have adopted the thoracoscopic approach and numerous reports described the outcomes of thoracoscopic EA/TEF repair [3]. Thoracoscopic repair provides several advantages including a magnified operative view, reduced chest wall morbidity, less postoperative pain, and more favorable cosmetic results compared with thoracotomy [4]. Unfortunately, the procedure remains technically challenging for most pediatric surgeons due to the rarity of these procedures, the particularly small working space in the neonatal thorax, and the demanding skills required for intracorporeal suturing of the esophageal anastomosis under single-lung ventilation [5,6].

Previous reports investigating the learning curve and postoperative outcomes focused mostly on high-volume centers with single experienced surgeons [5,6,7]. Furthermore, most learning curve analyses used arbitrary chronological period divisions rather than validated statistical methods such as the cumulative sum (CUSUM) technique, which has been shown to be a more sensitive method for the determination of the learning curve transition point [8]. Kim et al. [9] reported a learning curve of 10 cases using CUSUM analysis at a high-volume Korean center. However, data from developing MIS programs—particularly from Eastern European institutions with lower annual case volumes—remain scarce.

The aims of this study were: (1) to report the outcomes of thoracoscopic versus open EA/TEF repair during our institutional transition from open to thoracoscopic approach over a 10-year period; (2) to evaluate the learning curve using CUSUM analysis based on operative time; (3) to analyze the impact of long-gap esophageal atresia on thoracoscopic outcomes; and (4) to classify postoperative complications using the Clavien–Dindo grading system [10]. Each patient was assigned the single highest-grade complication for the primary analysis.

## 2. Materials and Methods

### 2.1. Patients and Data

At our center, the first case of thoracoscopic EA/TEF repair was performed on 29 October 2015. Between January 2015 and June 2026, all consecutive patients diagnosed with EA/TEF who underwent primary surgical repair at our institution were retrospectively reviewed. Patients operated at other centers who presented only for follow-up, evaluation, or secondary procedures such as fundoplication or esophageal dilation were excluded from the analysis (*n* = 11). The complete patient flow was as follows: 98 EA/TEF patients were assessed; 19 were excluded (11 operated at other centers and presented only for follow-up, 5 with insufficient records, 1 non-EA diagnosis, 1 converted-and-excluded complex case, and 1 excluded for a disproportionate complex late course), leaving 79 patients for analysis (28 thoracoscopic, 51 open). Patients with insufficient operative data (*n* = 5) and patients with non-EA diagnoses (*n* = 1) were also excluded. One patient who was converted from thoracoscopic to open approach and subsequently had a complex clinical course with multiple reinterventions over 5 years, culminating in aorto-esophageal fistula and death after esophageal stent placement, was excluded from the analysis due to the disproportionate effect on outcome measures: this patient underwent multiple reinterventions over a five-year period, ultimately developing an aorto-esophageal fistula and dying after esophageal stent placement. The inclusion of this single case would have substantially inflated the complication and mortality rates of the thoracoscopic group, reflecting a complex late trajectory rather than the perioperative outcomes under evaluation. A sensitivity analysis including this patient did not materially alter the primary findings. Finally, data from 79 patients were included: 28 underwent thoracoscopic repair and 51 open repair. The annual number of thoracoscopic cases is shown in Figure 1.

The following data were collected from hospital discharge summaries and operative records: gestational age, birth weight, sex, Gross classification, associated anomalies (cardiac, VACTERL), ASA score, preoperative respiratory support, time from birth to fistula ligation or gastrostomy, time from birth to anastomosis, surgical approach (thoracoscopic or open), operative time, intraoperative events (desaturation, hypercapnia, cardiac events), conversion to open, postoperative mechanical ventilation duration, anastomotic leak (confirmed by contrast esophagogram), leak grade and management, anastomotic stricture and number of dilation sessions, recurrent TEF, postoperative pneumothorax, chylothorax, pleural effusion, thorax drainage, sepsis, GERD, Nissen fundoplication, mortality and cause of death, total hospital stay, time to full oral feeding, contrast study timing, follow-up duration, and Clavien–Dindo complication grade.

This study was approved by the Institutional Review Board of “Grigore Alexandrescu” Emergency Hospital for Children (approval number: 29678) and the need for informed consent was waived due to the retrospective nature of the study.

### 2.2. Surgical Technique

The decision to perform thoracoscopic versus open repair was based on the operating surgeon’s judgment. All thoracoscopic procedures were performed by a single dedicated surgeon with prior experience in neonatal laparoscopic surgery, or under his direct supervision by surgeons in training. In contrast, open repairs were performed by multiple surgeons within the department over the study period, taking into account patient weight, gestational age, hemodynamic stability, severity of associated anomalies, and the anticipated gap length. No absolute contraindications to thoracoscopic approach were predefined; however, extreme prematurity (<28 weeks), severe hemodynamic instability requiring high-dose vasopressors, and active pulmonary hemorrhage were considered relative contraindications.

For thoracoscopic repair, the infant was placed in a left-lateral decubitus to three-quarter prone position (approximately 45° to 60° prone tilt) under general anesthesia with endotracheal intubation. Selective single-lung ventilation was attempted when feasible using mainstem bronchial intubation or bronchial blocker. Continuous monitoring of oxygen saturation (SpO_2_), end-tidal CO_2_, peak inspiratory pressures, and arterial blood gases was performed throughout the procedure. After insertion of a 5 mm 30-degree scope trocar just below the tip of the right scapula, the right thoracic cavity was insufflated with low-pressure carbon dioxide to create a controlled pneumothorax at 4–6 mmHg, which helped compress and maintain collapse of the ipsilateral lung. Two additional 3 mm working trocars were inserted: one at the right mid-axillary line along the scapular margin and one opposite the camera port.

The azygos vein was identified and divided using electrocautery or clips when it obstructed visualization or access to the esophageal segments. The distal esophagus was then dissected circumferentially from the trachea and the tracheoesophageal fistula was identified. The fistula was ligated using titanium clips, Hem-o-lok clips, or intracorporeal suture ligation with 3–0 braided polyglycolic absorbable sutures, depending on the fistula diameter and tissue quality. The fistula was then divided distal to the ligation site.

The proximal esophageal pouch was identified by asking the anesthesiologist to advance the nasogastric tube into the proximal pouch and was dissected circumferentially to obtain adequate length for a tension-free anastomosis. The esophageal anastomosis was performed using interrupted absorbable sutures (PDS or PGA 5-0/6-0) in a single-layer end-to-end fashion. The posterior wall sutures were placed first. After completion of the posterior row, the transanastomotic nasogastric tube (6–8 Fr) was advanced through the anastomosis into the distal esophagus and stomach under direct thoracoscopic visualization; correct positioning was confirmed by aspiration of gastric fluid through the tube. The anterior wall sutures were then placed to complete the anastomosis. The number of sutures varied from 8 to 14 depending on esophageal caliber. A 10 Fr chest tube was placed with the tip positioned adjacent to the anastomosis site.

Open repair was performed through a standard right posterolateral thoracotomy via the 4th or 5th intercostal space. The tracheoesophageal fistula was ligated exclusively by suture ligation without the use of clips, and the esophageal anastomosis was performed using the same interrupted absorbable suture technique as described for the thoracoscopic approach.

### 2.3. Management of Long-Gap Esophageal Atresia

For long-gap EA—defined as cases where primary anastomosis was not achievable due to excessive distance between esophageal segments—a staged approach was employed. An initial gastrostomy was performed (thoracoscopically or open) for enteral feeding access, followed by the Foker internal traction technique when the gap was deemed amenable to esophageal lengthening. The Foker technique was performed thoracoscopically in 5 cases and via open thoracotomy in 1 case. Pledgetted traction sutures were placed on the proximal and distal esophageal pouches and externalized through the chest wall. Gradual traction was applied over 3–7 days until the gap was reduced sufficiently for delayed primary anastomosis. The number of traction sessions ranged from 1 to 3. Pure esophageal atresia without distal TEF (Gross type A) was also classified as long-gap. Staged Foker procedures were not classified as Clavien–Dindo Grade IIIb reoperations, as they represent planned sequential interventions rather than reoperations for complications.

### 2.4. Preoperative Stabilization and Anesthetic Considerations

All patients underwent preoperative stabilization prior to surgical repair. This included hemodynamic stabilization with fluid resuscitation and, when necessary, vasopressor support (dopamine, noradrenaline). Preoperative respiratory support included continuous positive airway pressure (CPAP), high-flow nasal cannula (HFNC), or mechanical ventilation as indicated. Patients who were mechanically ventilated at admission were classified as requiring preoperative stabilization (ASA V). Echocardiography was performed in all patients to assess cardiac anatomy. Associated cardiac anomalies were classified as minor (patent foramen ovale, small patent ductus arteriosus, small atrial or ventricular septal defect, aberrant chordae) or major (tetralogy of Fallot, aortic coarctation, large ventricular septal defect, total anomalous pulmonary venous drainage, complete atrioventricular canal defect).

Intraoperative anesthetic challenges were prospectively documented and included: oxygen desaturation episodes (SpO_2_ < 85%), hypercapnia (EtCO_2_ > 55 mmHg), difficulty with single-lung ventilation, and cardiac events (bradycardia, arrhythmia). Temporary cessation of CO_2_ insufflation was performed when SpO_2_ dropped below 85%, with insufflation resumed once oxygen saturation recovered. This intermittent insufflation technique, while extending operative time, was essential for maintaining patient safety during the learning curve period.

### 2.5. Postoperative Management

Postoperative ventilator weaning was attempted as soon as hemodynamic and respiratory parameters allowed, typically within 24–72 h of the procedure. Extubation was performed when the infant demonstrated adequate spontaneous respiratory effort with acceptable blood gas values. Postoperative ventilation duration was calculated as the time from surgery to successful extubation.

Oral intake was restricted until a contrast esophagogram was performed to assess anastomotic integrity. In the absence of contrast extravasation, oral feeding was initiated with small-volume feeds and advanced gradually. The timing of oral feeding initiation varied considerably among patients and was strictly dependent on the moment of successful extubation, the presence or absence of anastomotic complications, and the overall clinical trajectory. Patients with prolonged mechanical ventilation, anastomotic leak requiring conservative management, or significant respiratory complications experienced substantially delayed feeding initiation. Hospital stay was defined as the number of days from admission to discharge of the surgical admission (the admission during which the anastomosis was performed).

### 2.6. Definitions of Outcomes

Anastomotic leak was defined strictly as contrast extravasation on postoperative esophagogram—either visualization of a fistulous tract from the anastomosis site, or presence of contrast material in the pleural cavity or mediastinum. Patients whose esophagogram showed no contrast extravasation were classified as having no leak, even if clinical suspicion existed. Leaks were graded as minor (small fistulous tract without clinical consequence) or major (contrast in the pleural cavity or mediastinum). Leak management was classified as conservative (antibiotics, NPO, TPN), chest drainage, or surgical reintervention (re-anastomosis or anastomotic reinforcement).

Anastomotic stricture was defined as esophageal narrowing causing dysphagia or feeding difficulty, confirmed endoscopically and requiring balloon dilation. Each endoscopic dilation session requiring a separate hospital admission was counted as one dilation episode. Recurrent TEF was defined as recurrence of the tracheoesophageal communication after primary repair, confirmed by contrast study or bronchoscopy.

Complications were classified according to the Clavien–Dindo system [10]: Grade I (postoperative ventilatory disturbances stabilized with supportive care only), Grade II (pharmacological hemodynamic support with vasopressors—dopamine, noradrenaline, dobutamine—or transfusion of blood products: packed red blood cells, fresh frozen plasma, platelets), Grade IIIa (bedside thoracic drainage for pneumothorax, anastomotic leak, chylothorax, or pleural effusion), Grade IIIb (surgical reintervention under general anesthesia for anastomotic complications—re-anastomosis, reinforcement, or refashioning), Grade IV (single- or multi-organ failure—renal, cardiac, hepatic, or multiorgan dysfunction syndrome—in a surviving patient), and Grade V (death of the patient).

### 2.7. Statistical Analysis

Descriptive statistics and data management were performed using Microsoft Excel (Microsoft Corporation, Redmond, WA, USA). Inferential statistical analysis was performed using R version 4.3.2 (R Foundation for Statistical Computing, Vienna, Austria). A *p*-value < 0.05 was considered statistically significant, and confidence intervals were set at the 95% level. Continuous variables are presented as mean ± standard deviation (SD), median, and range and were compared using the Mann–Whitney U test. For skewed variables, median with interquartile range (IQR) is also reported due to the non-normal distribution of most variables in the small sample. Categorical variables are presented as number of cases and percentage (%) and were compared using Fisher’s exact test. Odds ratios (ORs) with 95% confidence intervals (CIs) were calculated for binary outcomes. All *p*-values are reported regardless of statistical significance to ensure transparency and avoid publication bias.

Follow-up was calculated in days from the surgery date to the date of last recorded hospital discharge. Patients with a single hospital admission were classified as having no follow-up in the institutional database. Among the 42 patients (53%) with institutional follow-up, the median follow-up was 394 days (IQR 236–791 days); 38 patients (90.5%) had at least 3 months, 32 (76.2%) at least 6 months, and 22 (52.4%) at least 12 months of follow-up. Patients without institutional follow-up were predominantly those managed acutely who returned to referring institutions, so missingness is likely non-random.

### 2.8. Cumulative Sum Method and Learning Curve Analysis

A conventional binary cumulative sum (CUSUM) method was used to evaluate the learning curve for thoracoscopic EA/TEF repair [11,12]. The CUSUM technique is a graphical representation of the outcome trend in a series of consecutive procedures performed over time. A binary outcome is required for CUSUM analysis: in this study, surgical failure was defined as operative time exceeding the mean for the entire thoracoscopic cohort. The converted patient was excluded from the study cohort and was not included as a CUSUM observation.

The 28 thoracoscopic cases were ordered chronologically and assigned sequential case numbers. In the CUSUM plot, procedures were plotted sequentially from left to right. The graph ascended for every procedure where the operative time exceeded the mean (surgical failure) and descended for every procedure where the time was below the mean (surgical success). The learning curve was identified as the point where the CUSUM curve peaked and began a sustained decline, indicating that subsequent procedures were consistently completed in less than the mean time [9].

For chronological phase analysis, the 28 cases were divided into three phases of approximately equal size: Phase 1—Early (cases 1–9, October 2015–December 2022), Phase 2—Intermediate (cases 10–18, January 2023–June 2024), and Phase 3—Proficient (cases 19–28, July 2024–June 2026). Operative time differences between Phase 1 and Phase 3 were tested using the Mann–Whitney U test.

### 2.9. Long-Gap Subgroup Analysis

Within the thoracoscopic group, patients were stratified into long-gap (*n* = 8) and short-gap (*n* = 20) subgroups. Long-gap was defined as: (a) Foker internal traction technique used, (b) pure esophageal atresia without distal TEF (Gross type A), or (c) gap length requiring staged repair (gastrostomy followed by delayed anastomosis). Short-gap was defined as cases where primary single-stage anastomosis was performed. Postoperative outcomes including leak, stricture, pneumothorax, hospital stay, ventilation duration, and mortality were compared between subgroups.

## 3. Results

Demographics and surgical outcomes of the 79 patients are shown in Table 1. The mean birth weight was 2672 ± 661 g and the mean gestational age was 36.9 ± 2.2 weeks. The mean operative time was 2.9 ± 1.2 h in the thoracoscopic group versus 2.3 ± 1.2 h in the open group (*p* = 0.036). The median postoperative ventilation was 168 h (range 48–456) in the thoracoscopic group. Anastomotic leak confirmed by contrast esophagogram occurred in 9/28 (32.1%) thoracoscopic and 7/51 (13.7%) open patients (*p* = 0.137, OR = 2.51, 95% CI 0.80–7.89). Of the 16 total leaks, 8 (50.0%) were managed conservatively, 2 (12.5%) with chest drainage, and 6 (37.5%) required surgical reintervention. Anastomotic stricture requiring balloon dilation occurred in 3/28 (10.7%) versus 3/51 (5.9%; *p* = 0.660). No cases of recurrent TEF occurred. Mortality was 2/28 (7.1%) versus 3/51 (5.9%; *p* = 1.000).

Clavien–Dindo classification is shown in Table 2. Grade IIIa was the most frequent complication in the thoracoscopic group (64.3%), driven by pneumothorax requiring drainage. Grade IIIb (reintervention) occurred in 3.6% thoracoscopic vs. 7.8% open patients. No Grade IV events occurred.

The CUSUM plot based on operative time is shown in Figure 2. The curve ascended during the first 10 procedures, peaked at case 1 and subsequently declined continuously without further reascension, indicating that the learning curve was identified at approximately case 15. Table 3 shows outcomes by learning curve phase. Operative time decreased significantly from Phase 1 to Phase 3 (3.4 ± 1.3 h vs. 2.5 ± 0.8 h; *p* = 0.034). No strictures were observed in Phase 3 (Figure 3).

Within the thoracoscopic group, 8 patients were classified as long-gap and 20 as short-gap (Table 4). Long-gap patients had longer hospital stays (66.1 vs. 29.8 days; *p* = 0.005) and significantly higher stricture rates in this exploratory subgroup analysis (37.5% vs. 0%; *p* = 0.017). The three long-gap patients with stricture required 11 total dilation sessions. The operative time recorded for long-gap cases reflects only the individual delayed anastomosis rather than the entire staged pathway (gastrostomy, traction procedures, and definitive anastomosis); the shorter per-procedure time therefore does not indicate a technically simpler operation and is not directly comparable. Kaplan–Meier estimated survival was 87.5% (long-gap) vs. 95.0% (short-gap; *p* = 0.497) (Figure 4).

## 4. Discussion

Thoracoscopic repair of EA/TEF is still a technical challenge demanding a high level of surgical skill. At our center, thoracoscopic repair was introduced in 2015 and progressively adopted as the preferred approach, reaching 7–8 cases per year by 2024. This gradual transition allowed the surgical team to accumulate experience in patient positioning, anesthetic management of single-lung ventilation, intracorporeal suturing, and postoperative care protocols specific to thoracoscopic EA repair.

The CUSUM analysis in our series revealed a learning curve of approximately 15 cases, with operative time decreasing from 3.4 h (Phase 1) to 2.5 h (Phase 3; *p* = 0.034). Kim et al. reported a learning curve of 10 cases using the same methodology at Samsung Medical Center, with a mean operative time of 144 ± 65 min [9]. The longer learning curve at our center likely reflects the lower annual case volume (1–3 cases/year in 2015–2022 vs. 5–10 cases/year at high-volume centers). Similarly, Hiradfar et al. [13] reported a reduction in conversion rates from 58.3% to 35.7% after the first 10 thoracoscopic cases, further supporting the 10–15 case threshold for initial proficiency. Volume concentration appears to be a critical factor in accelerating surgical proficiency. Importantly, van der Zee et al. [6] demonstrated at the Wilhelmina Children’s Hospital (Utrecht) that once an institutional thoracoscopic program matures, complication rates—including anastomotic leak, stricture, and recurrent fistula—decrease significantly even when procedures are predominantly performed by less experienced surgeons, young staff members, and fellows under senior mentorship. This finding suggests that the learning curve for thoracoscopic EA repair is institutional rather than purely individual. In our program, a single surgeon performed or directly supervised all 28 thoracoscopic procedures, while trainees progressively participated under mentorship. The open repairs, by contrast, were performed by several surgeons within the department, reflecting the broader institutional practice prior to the adoption of thoracoscopy: it depends not only on the operating surgeon’s manual dexterity but also on the cumulative experience of the entire surgical team, including standardized anesthetic protocols, dedicated nursing staff, protocolized postoperative management, and the availability of an experienced mentor for intraoperative guidance. Our own experience mirrors this pattern: the reduction in operative time and the trend toward fewer complications in Phase 3 coincided with the maturation of our institutional protocols and the growing confidence of our anesthetic and neonatal intensive care teams in managing the specific challenges of neonatal thoracoscopy.

The overall complication rate in our thoracoscopic group was higher than in the open group, with Clavien–Dindo Grade ≥ III complications occurring in 75.0% versus 54.9% of patients. This finding deserves nuanced interpretation. First, as a retrospective study spanning over a decade, complications in the earlier open era (2015–2018) may have been underreported or inconsistently documented compared to the thoracoscopic era (2019–2026), where standardized follow-up protocols and systematic contrast studies were established. Second, and more importantly, the majority of complications in the thoracoscopic group were managed conservatively without requiring surgical reintervention. Of the 8 anastomotic leaks in the thoracoscopic group, 6 (75.0%) were treated with conservative management (antibiotics, nil per os, parenteral nutrition) or simple pleural drainage, with spontaneous healing of the leak confirmed on follow-up contrast studies [14,15]. Only 2 (25.0%) required reoperation. Similarly, postoperative pneumothorax—the most common Grade IIIa complication—was managed with bedside chest tube drainage and resolved without further intervention. These findings are consistent with published learning curve series from other developing MIS programs, where higher initial complication rates are expected but tend to decrease with institutional experience, and where the vast majority of leaks heal spontaneously with adequate drainage and nutritional support [14,15,16,17]. The capacity to manage complications conservatively rather than with repeat surgery represents a meaningful clinical advantage, as it avoids the morbidity associated with redo thoracotomy in the neonatal period.

The higher pneumothorax rate in the thoracoscopic group (53.6% vs. 17.6%; *p* = 0.002; OR = 5.38) is multifactorial. It partly reflects the technical consequence of CO_2_ insufflation and managed lung collapse during thoracoscopy and partly correlates with the higher leak rate, as several pneumothoraxes were secondary to air leak through the anastomotic breach. The Clavien–Dindo analysis confirmed that Grade IIIa complications were the predominant morbidity in the thoracoscopic group (64.3%), while Grade IIIb (reoperation) was lower (3.6% vs. 7.8%), suggesting that thoracoscopic repair generates more minor procedural complications without increasing the risk of major adverse outcomes.

Our anastomotic leak rate of 32.1% in the thoracoscopic group is higher than the 6% reported by Kim et al. [9] and the 10–15% reported by Rothenberg [18] and consistent with the pooled thoracoscopic leak rate in the systematic review by Zani et al. [19]. However, our strict radiological definition—contrast extravasation on esophagogram—may capture subclinical leaks that resolve spontaneously. Indeed, 46.7% of leaks were managed conservatively without surgical reintervention. Furthermore, leak rates showed a downward trend across phases (33.3% in Phases 1–2 vs. 20.0% in Phase 3), suggesting gradual improvement in anastomotic technique that had not yet reached statistical significance in our sample size.

GERD documentation was significantly higher in the thoracoscopic group (96.4% vs. 41.2%; *p* < 0.001). We interpret this as a surveillance bias: patients operated in the recent thoracoscopic era underwent systematic endoscopic follow-up with pH monitoring, while many open patients from the earlier era did not have standardized GERD assessment protocols.

The initiation and progression of oral feeding after EA/TEF repair followed a standardized stepwise protocol at our institution. After confirmation of anastomotic integrity on contrast esophagogram, oral feeding was initiated with 5% glucose solution administered in small volumes by bottle. Once glucose tolerance was established, transition to a milk formula was undertaken. In our cohort, anti-reflux (AR) thickened formulas were the predominant choice for initial enteral nutrition reflecting the near-universal prevalence of gastroesophageal reflux in this population. For patients with complicated postoperative courses, particularly those with anastomotic leak managed conservatively, prolonged ventilation, or long-gap repairs, an elemental formula was introduced first, followed by gradual transition to an anti-reflux formula once enteral tolerance was confirmed. Breast milk was used when available, either exclusively or combined with AR formula supplementation, and was documented in 5 patients (6.3%). Expressed breast milk was preferred over direct breastfeeding in the early postoperative period to allow precise volume control and monitoring for aspiration or reflux during feeds [20,21].

The achievement of full enteral feeding was not solely determined by surgical or medical factors but was also significantly influenced by the process of maternal adaptation. Once the infant was clinically stable and tolerating bottle feeds under nursing supervision in the neonatal intensive care unit, the mother was progressively introduced into the feeding process. Initially, maternal feeding sessions were conducted in the NICU under direct supervision of the neonatology team, who observed for signs of aspiration, choking, or significant reflux episodes. Subsequently, the infant was transferred to the mother’s room, with frequent neonatologist visits at mealtimes to monitor feeding technique and reassure the mother. In our experience, the most common maternal concern was the fear of aspiration and choking during feeds, particularly in the context of visible gastroesophageal reflux and the infant’s occasional coughing or desaturation during swallowing. Parental anxiety regarding reflux management frequently prolonged the transition to independent feeding and, consequently, hospital discharge. This observation is consistent with published data demonstrating that feeding difficulties and parental anxiety are among the most significant determinants of prolonged hospitalization and impaired quality of life in EA/TEF families [20,21,22].

Our stricture rate of 10.7% is lower than the 32% reported by Kim et al. [9]. However, the subgroup analysis revealed a striking difference: long-gap thoracoscopic patients had a 37.5% stricture rate compared to 0% in short-gap patients (*p* = 0.017). All three stricture patients had undergone Foker traction before anastomosis, suggesting that anastomotic tension may be an important determinant of stricture risk regardless of the surgical approach. Notably, none of our long-gap patients required esophageal replacement surgery (colonic or gastric interposition). The adoption of the Foker internal traction technique, performed thoracoscopically in the majority of cases, allowed preservation of the native esophagus in all long-gap patients. This represents a significant shift from the traditional esophagostomy-based approach, which frequently committed patients to replacement procedures with their attendant long-term morbidity [18]. The avoidance of esophageal replacement, despite the higher stricture rate in long-gap cases, supports the feasibility of the traction-based strategy for esophageal preservation in long-gap EA. These three patients required 11 total dilation sessions, representing a significant long-term morbidity burden.

The evolution of surgical techniques for esophageal atresia over the past two decades reflects a paradigm shift from esophageal replacement toward native esophagus preservation [23,24]. Historically, long-gap EA was managed with cervical esophagostomy and gastrostomy, committing patients to esophageal replacement using colonic interposition, gastric pull-up, or jejunal interposition—procedures associated with significant long-term morbidity including graft dysfunction, anastomotic stricture at the cervical level, dumping syndrome, chronic aspiration, and lifelong gastroesophageal reflux [25,26]. Contemporary evidence increasingly supports that preserving the native esophagus, whenever technically feasible, yields superior functional outcomes in terms of swallowing physiology, esophageal motility, and long-term quality of life [24,27]. The Foker internal traction technique and its minimally invasive variants have emerged as the preferred strategy for native esophagus preservation in long-gap EA, enabling delayed primary anastomosis after tension-induced esophageal growth [28,29]. At our center, the transition from esophagostomy-based management to the Foker technique coincided with the adoption of the thoracoscopic approach, allowing traction procedures to be performed minimally invasively in the majority of long-gap cases. None of the long-gap patients in our thoracoscopic series required esophageal replacement surgery, supporting the feasibility of this approach even in developing MIS centers. The American Pediatric Surgery Association (APSA) evidence-based guidelines now recommend delayed primary anastomosis as the preferred initial approach for long-gap EA, with esophageal replacement reserved for cases where all attempts at native esophagus preservation have failed [30]. The combination of minimally invasive access and the Foker technique represents the current frontier in long-gap EA management, enabling esophageal preservation with reduced chest wall morbidity [31,32].

Emerging adjunctive techniques such as intramural botulinum toxin injection during esophageal traction have been described in the literature, although current evidence remains limited and their potential application in thoracoscopic Foker procedures warrants further investigation.

A critical area requiring future investigation is the optimization of ventilatory strategies during neonatal thoracoscopic surgery. The interplay between CO_2_ insufflation, single-lung ventilation, and the immature neonatal respiratory physiology creates a uniquely challenging anesthetic environment [33,34]. In our experience, intraoperative desaturation episodes (SpO_2_ < 85%) requiring temporary cessation of CO_2_ insufflation and respiratory re-equilibration were frequent, particularly during the early learning curve period. These events were managed by exsufflation of the thoracic cavity, allowing bilateral lung re-expansion and hemodynamic stabilization before resuming the procedure. While these episodes were resolved rapidly and without apparent clinical consequences, they were not systematically documented in the operative records, representing a significant gap in our data collection. The hemodynamic and cerebral oxygenation effects of intermittent CO_2_ insufflation and desaturation in the neonatal brain remain incompletely understood. Ijsselstijn et al. [33] demonstrated that intrathoracic CO_2_ insufflation caused reversible decreases in SaO_2_ and pH with increases in paCO_2_, while cerebral oxygenation measured by near-infrared spectroscopy remained within normal limits. However, the cumulative impact of repeated desaturation-reinsufflation cycles on the developing brain has not been studied. Recent work by Yang et al. [35] suggests that pressure-controlled ventilation with volume guarantee (PCV-VG) may improve oxygenation and reduce barotrauma during neonatal thoracoscopic EA repair, while Zheng et al. [36] have explored the use of high-frequency ventilation as an alternative strategy. Future prospective studies should focus on standardized documentation of intraoperative ventilatory events, comparative evaluation of ventilation modes (conventional vs. PCV-VG vs. high-frequency), optimal insufflation pressures for neonates of varying gestational ages, and the long-term neurodevelopmental impact of intraoperative hypoxic and hypercapnic episodes.

There are several limitations to this study. First, the retrospective design introduces inherent selection bias. Second, the sample size (*n* = 79, with 28 thoracoscopic cases) limits statistical power for detecting differences in rare outcomes such as mortality and recurrent TEF. Third, the thoracoscopic and open groups are not contemporaneous—most open cases preceded the thoracoscopic era, and the open group functions as a historical institutional comparator rather than a concurrent control, introducing temporal confounding. Fourth, postoperative ventilation data were available for only 51% of patients. Fifth, follow-up data were available for 53% of patients. With only 28 thoracoscopic cases, this study is underpowered to detect differences in rare outcomes, and all subgroup comparisons should be considered exploratory rather than confirmatory; complete binary outcome data (leak, stricture, pneumothorax, mortality) were available for all 79 patients. Finally, the CUSUM analysis was based solely on operative time; future studies should incorporate composite outcome measures including complication rates.

## 5. Conclusions

Thoracoscopic repair of EA/TEF is feasible in a developing MIS center with acceptable early outcomes. The CUSUM-based learning curve was approximately 15 cases, longer than the 10 cases reported at high-volume centers, likely reflecting our lower annual case volume. Long-gap thoracoscopic repair carries a significantly higher stricture risk (37.5% vs. 0%; *p* = 0.017) and requires longer hospitalization. In this underpowered analysis, no statistically significant difference in mortality was observed between groups. The transition from open to thoracoscopic EA repair requires sustained institutional commitment, careful patient selection, progressive case volume concentration, and systematic complication monitoring using standardized classification systems.

## Figures and Tables

**Figure 1:**
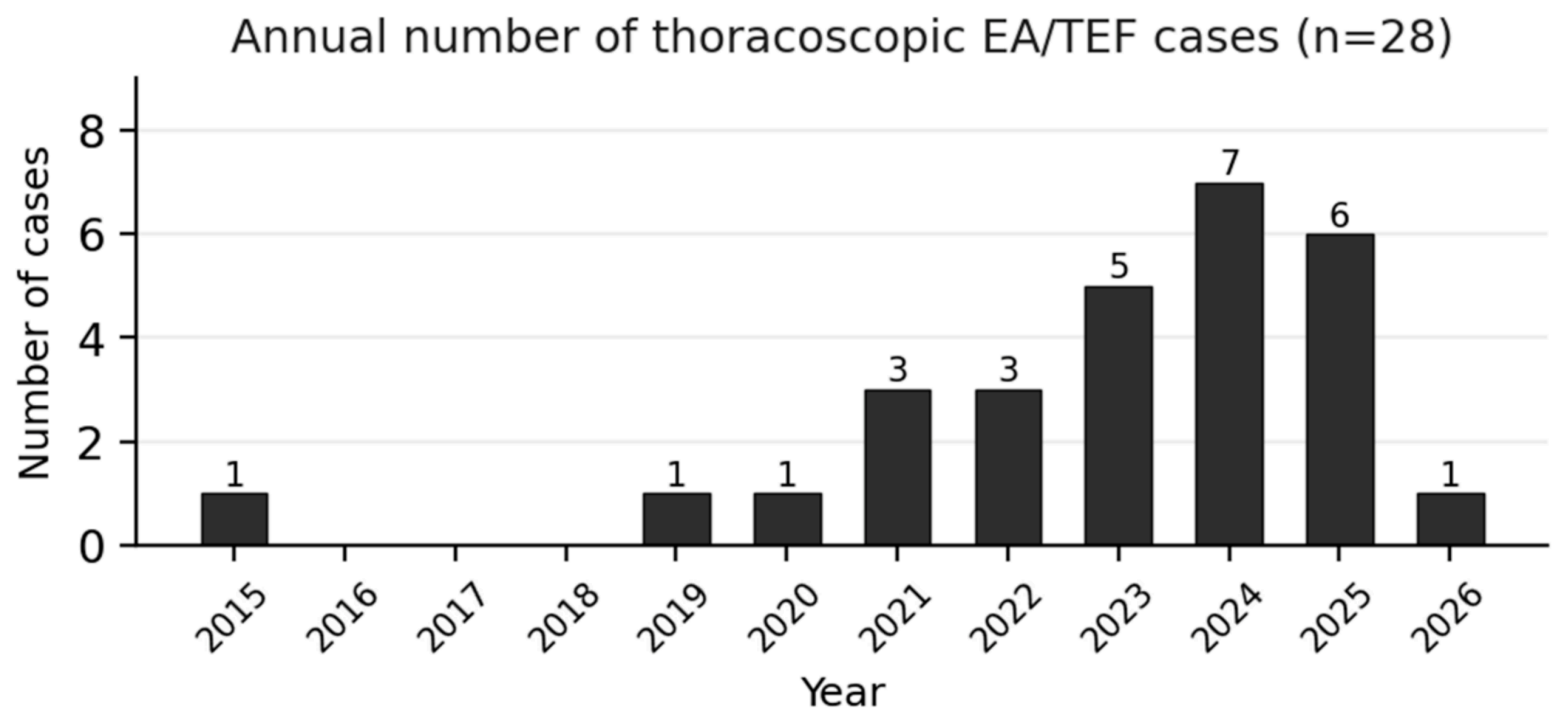
Annual number of thoracoscopic cases.

**Figure 2:**
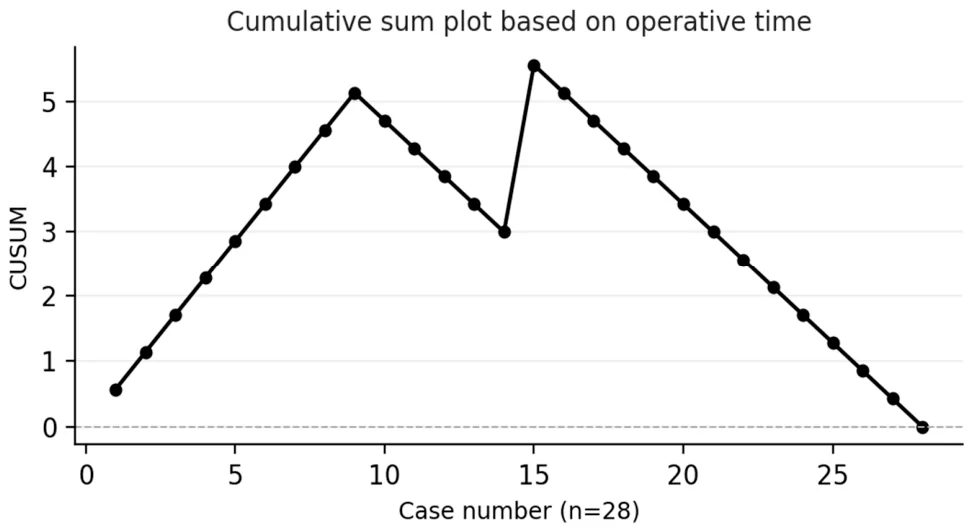
Cumulative sum plot based on operative time in our center.

**Figure 3:**
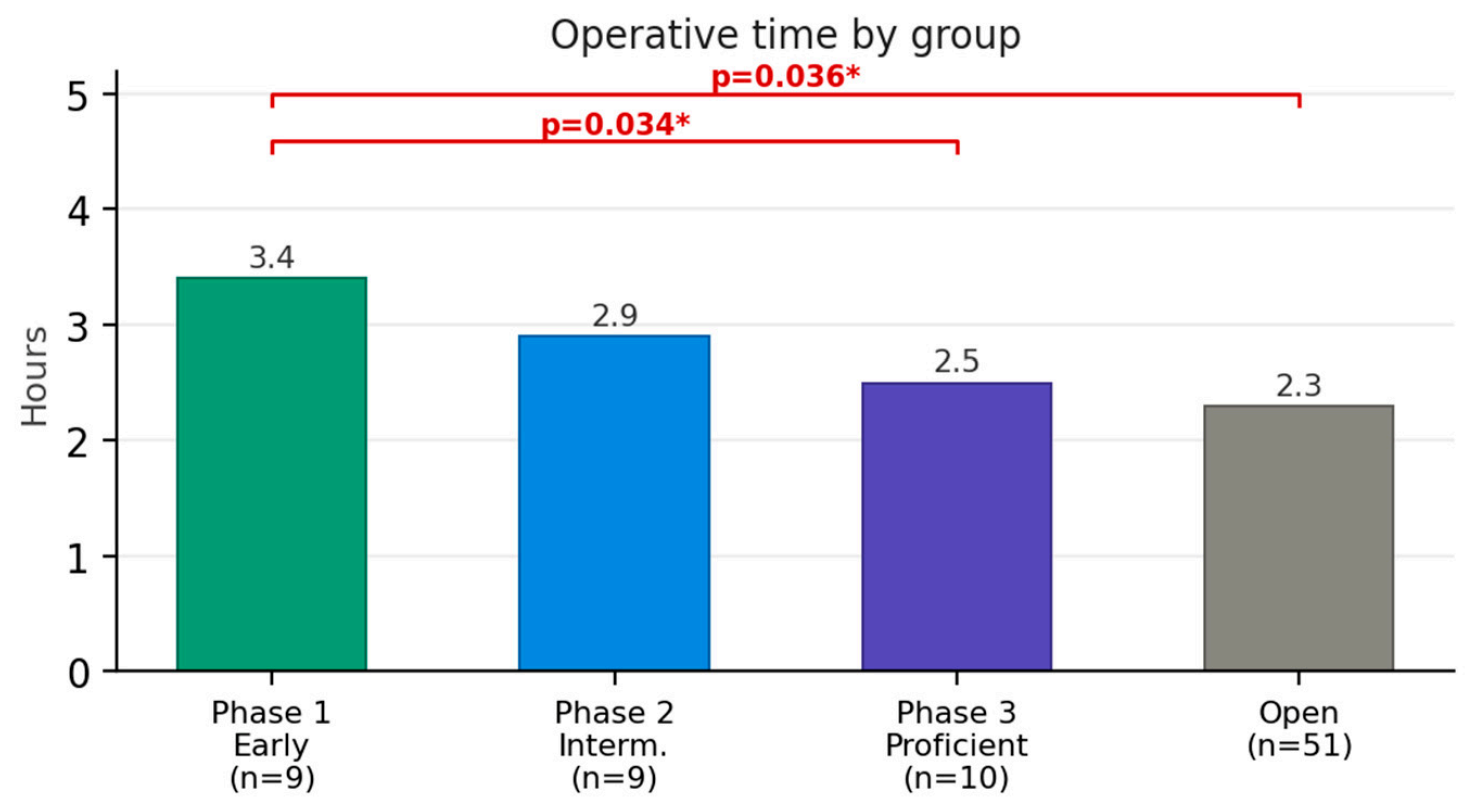
Operative time by group. * *p* = 0.034 Phase 1 vs. Phase 3; *p* = 0.036 thoracoscopic vs. open.

**Figure 4:**
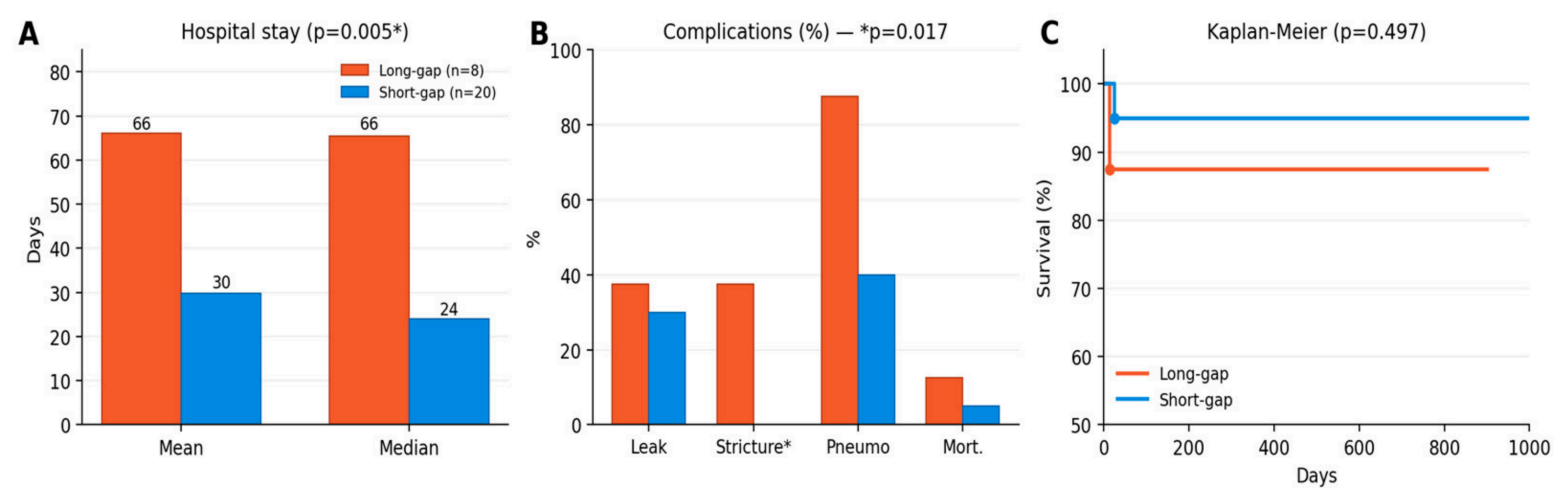
Long-gap vs. short-gap: (**A**) hospital stay, (**B**) complications (* stricture *p* = 0.017), (**C**) Kaplan–Meier survival.

**Table 1:** Patient demographics and surgical outcomes.

Variables	Thoracoscopic (*n* = 28)	Open (*n* = 51)	*p*
Male:female	14:14	26:25	1.000
GA (weeks), mean ± SD	36.9 ± 2.0	36.9 ± 2.3	0.363
Birth weight (g)	2732 ± 607	2618 ± 709	0.535
Prematurity (<37 w), *n* (%)	12 (42.9)	11 (21.6)	0.069
Gross type C, *n* (%)	26 (92.9)	47 (92.2)	1.000
Long-gap EA, *n* (%)	8 (28.6)	12 (23.5)	0.787
Foker technique, *n* (%)	5 (17.9)	1 (2.0)	0.019 *
Major cardiac anomaly, *n* (%)	3 (10.7)	4 (7.8)	0.694
ASA V, *n* (%)	8 (28.6)	10 (19.6)	0.413
Operative time (h)	2.9 ± 1.2	2.3 ± 1.2	0.036 *
Postop. MV (h)	170.6 ± 99.2	152.6 ± 76.2	0.568
Hospital stay (days)	40.2 ± 25.7	29.2 ± 15.8	0.197
Anastomotic leak, *n* (%)	9 (32.1)	7 (13.7)	0.137
Conservative	6 (21.4)	2 (3.9)	
Chest drainage	1 (3.6)	1 (2.0)	
Reintervention	2 (7.1)	4 (7.8)	
Stricture, *n* (%)	3 (10.7)	3 (5.9)	0.660
Recurrent TEF	0 (0)	0 (0)	1.000
Pneumothorax, *n* (%)	15 (53.6)	9 (17.6)	0.002 *
GERD, *n* (%)	27 (96.4)	21 (41.2)	<0.001 *
Nissen, *n* (%)	3 (10.7)	1 (2.0)	0.125
Mortality, *n* (%)	2 (7.1)	3 (5.9)	1.000

GA, gestational age; MV, mechanical ventilation; GERD, gastroesophageal reflux. Data: mean ± SD or *n* (%). * *p* < 0.05.

**Table 2:** Clavien–Dindo classification.

Grade	Thoracoscopic (*n* = 28)	Open (*n* = 51)	Total (*n* = 79)
None	0 (0)	6 (11.8)	6 (7.6)
I	4 (14.3)	8 (15.7)	12 (15.2)
II	3 (10.7)	9 (17.6)	12 (15.2)
IIIa	18 (64.3)	21 (41.2)	39 (49.4)
IIIb	1 (3.6)	4 (7.8)	5 (6.3)
IV	0 (0)	0 (0)	0 (0)
V	2 (7.1)	3 (5.9)	5 (6.3)
≥III	21 (75.0)	28 (54.9)	49 (62.0)

Data: *n* (%).

**Table 3:** Learning curve phase analysis.

Variables	Phase 1 Early (*n* = 9)	Phase 2 Interm. (*n* = 9)	Phase 3 Proficient (*n* = 10)	Open (*n* = 51)	*p* *
Op. time (h)	3.4 ± 1.3	2.9 ± 1.4	2.5 ± 0.8	2.3 ± 1.2	0.034 *
Ventilation (h)	228.7 ± 126.9	133.7 ± 90.7	157.7 ± 66.2	152.6 ± 76.2	0.568
Hosp. stay (d)	40.2 ± 28.7	41.7 ± 26.5	38.8 ± 24.9	29.2 ± 15.8	0.935
Leak, *n* (%)	3 (33.3)	3 (33.3)	2 (20.0)	7 (13.7)	—
Stricture	1 (11.1)	2 (22.2)	0 (0)	3 (5.9)	—
Mortality	1 (11.1)	0 (0)	1 (10.0)	3 (5.9)	—

* *p*-value: Mann–Whitney U test, Phase 1 vs. Phase 3 only (open group shown for reference). Data: mean ± SD or *n* (%).

**Table 4:** Thoracoscopic subgroup: long-gap vs. short-gap.

Variables	Long-Gap (*n* = 8)	Short-Gap (*n* = 20)	*p*
Op. time (h)	2.3 ± 1.9	3.2 ± 0.7	0.004 *
Ventilation (h)	115.0 ± 52.8	184.5 ± 104.3	0.059
Hosp. stay (d)	66.1 ± 27.9	29.8 ± 16.0	0.005 *
Gastrostomy, *n* (%)	6 (75.0)	0 (0)	<0.001 *
Foker, *n* (%)	7 (87.5)	0 (0)	<0.001 *
Leak, *n* (%)	3 (37.5)	6 (30.0)	1.000
Stricture, *n* (%)	3 (37.5)	0 (0)	0.017 *
Pneumothorax	7 (87.5)	8 (40.0)	1.000
Mortality	1 (12.5)	1 (5.0)	0.497

* *p* < 0.05. Long-gap = Foker, Gross A, or staged repair.

## Data Availability

The data presented in this study are available on request from the corresponding author. The data are not publicly available due to patient privacy considerations.
